# The Surgeon's Vade Mecum: A Hand-Book of the Principles and Practice of Surgery

**Published:** 1839-10

**Authors:** 


ART. VI.—*The Surgeon's Vade Mecum: a Hand-book of the Principles and Practice of Surgery. Illustrated with numerous Wood Engravings.* By ROBERT DRUITT, M.R.C.S. *London*, 1839. Post 8vo, pp. 429.

SUCH of our readers as recollect the fierce onslaught we made on the writers of guide books, in our Eleventh Number, (*Brit. and Foreign Med. Review*, vol. VI. p. 202,) need not be informed how jealous we are of everything that is calculated to draw our younger brothers from the straight and narrow path of legitimate study to the royal road—the *facilis descensus Averni* of the grinder. One of the works there condemned was by the author of the present; but we had no fault to find with it, except on the general principle. The small volume before us has somewhat higher aims, being intended not so much as a help for the student as a prompter to the practitioner. It contains "a familiar account of the nature and treatment of the various diseases that are commonly assigned to the surgeon's care;" and will, no doubt, be found useful to those whose knowledge is not always ready at command. The author shows himself well acquainted with the most approved views both of pathology and practice; and the general arrangement of the subjects and the style in which the information is conveyed are particularly good. We have noticed some errors and more omissions; but, on the whole, the work is very creditable to the author, and we recommend it to the advanced pupil and to the young surgeon as a useful index to the great book of surgical knowledge.

